# Clinical classification of JIA in Albania

**DOI:** 10.1186/1546-0096-12-S1-P186

**Published:** 2014-09-17

**Authors:** Aurel Vula, Genti Xhelilaj, Loreta Teneqexhi

**Affiliations:** 1 UHC “Mother Tereza” Tirana, Albania; 2 Department of Public Health, University of Medicine, Tirana, Albania

## Introduction

Juvenile idiopathic arthritis (JIA) is a group of heterogeneous disorders with different disease manifestations among various populations. In our country is the first study made for the clinical patterns AIJ. In this paper presented AIJ clinical patterns encountered in University Hospital Center "Mother Teresa" in Tirana for the period July 2012-March 2014.

## Objectives

To identify clinical patterns of JIA in our country. Design of therapeutic strategies based on relevant clinical model.

## Methods

Hospital records of patients with a diagnosis of chronic arthritis with onsen at the age of 16 years or less presenting to University Hospital Center, “Mother Teresa”, Tirana for the periods July 2010 – March 2014 were retrospectively reviewed and reclassified as juvenile idiopathic arthritis (JIA) based on the International League of Associations for Rheumatology (ILAR) diagnostic criteria.

## Results

In total, 102 patients with chronic arthritis of onset at age 16 years or less were evaluated over these periods at the hospital. Of these, 44 could further by analyzed by ILAR JIA criteria. The average age at disease onset among the 44 JIA patients was 8.70 years (range: 1-15 years) with average age at first visit to hospital being 11.3 years (range: 2 to 15 years) and with a female to male ratio of 1.2:1. Polyarticular rheumatoid factor negative JIA, at 38.6%, was the most frequent type of chronic arthritis encountered. Oligoarthritis was found in 34.1%, while 9.1% and 11.3% were polyarticular rheumatoid factor positive and systemic JIA, respectively. Enthesitis - related arthritis was found in 4.5% and only 2.2% were determined to have psoriatic arthritis among this population.

## Conclusion

JIA is a predominantly rheumatoid factor negative polyarticular disease in Albania. Late presentation is an issue with major implications for educational input and resource acquisition. There is need to elucidate the genetics and environmental factors of JIA in this region.

## Disclosure of interest

None declared.

